# Vitamin A regulates tissue-specific organ remodeling in diet-induced obesity independent of mitochondrial function

**DOI:** 10.3389/fendo.2023.1118751

**Published:** 2023-02-20

**Authors:** Ivanna Shymotiuk, Natali Froese, Christopher Werlein, Lea Naasner, Malgorzata Szaroszyk, Mark P. Kühnel, Danny D. Jonigk, William S. Blaner, Adam R. Wende, E. Dale Abel, Johann Bauersachs, Christian Riehle

**Affiliations:** ^1^ Department of Cardiology and Angiology, Hannover Medical School, Hannover, Germany; ^2^ Institute of Pathology, Hannover Medical School, Hannover, Germany; ^3^ Biomedical Research in End-stage and Obstructive Lung Disease Hannover (BREATH), German Lung Research Centre (DZL), Hannover, Germany; ^4^ Department of Medicine, College of Physicians and Surgeons, Columbia University, New York, NY, United States; ^5^ Division of Molecular and Cellular Pathology, Department of Pathology, University of Alabama at Birmingham, Birmingham, AL, United States; ^6^ Department of Medicine, David Geffen School of Medicine and University of California, Los Angeles (UCLA), Health, Los Angeles, CA, United States

**Keywords:** mitochondria, vitamin A, diet-induced obesity, type 2 diabetes, liver, kidney, skeletal muscle

## Abstract

**Background:**

Perturbed mitochondrial energetics and vitamin A (VitA) metabolism are associated with the pathogenesis of diet-induced obesity (DIO) and type 2 diabetes (T2D).

**Methods:**

To test the hypothesis that VitA regulates tissue-specific mitochondrial energetics and adverse organ remodeling in DIO, we utilized a murine model of impaired VitA availability and high fat diet (HFD) feeding. Mitochondrial respiratory capacity and organ remodeling were assessed in liver, skeletal muscle, and kidney tissue, which are organs affected by T2D-associated complications and are critical for the pathogenesis of T2D.

**Results:**

In liver, VitA had no impact on maximal ADP-stimulated mitochondrial respiratory capacity (V_ADP_) following HFD feeding with palmitoyl-carnitine and pyruvate each combined with malate as substrates. Interestingly, histopathological and gene expression analyses revealed that VitA mediates steatosis and adverse remodeling in DIO. In skeletal muscle, VitA did not affect V_ADP_ following HFD feeding. No morphological differences were detected between groups. In kidney, V_ADP_ was not different between groups with both combinations of substrates and VitA transduced the pro-fibrotic transcriptional response following HFD feeding.

**Conclusion:**

The present study identifies an unexpected and tissue-specific role for VitA in DIO that regulates the pro-fibrotic transcriptional response and that results in organ damage independent of changes in mitochondrial energetics.

## Introduction

The number of patients suffering from diabetes mellitus is dramatically increasing with a current estimate that 1 in 10 people have diabetes worldwide reaching a total number of 537 million adults (www.idf.org). Most of these patients suffer from type 2 diabetes (T2D). Peripheral insulin resistance is a cardinal feature of T2D. Mitochondrial dysfunction is associated with insulin resistance and T2D (1, 2). Landmark studies reported impaired mitochondrial capacity in skeletal muscle from insulin resistant individuals (3) and T2D patients (4). Similarly, mitochondrial dysfunction has been reported in various tissues from rodent models with diet-induced obesity (DIO) and T2D (5, 6). A close interplay between insulin resistance and mitochondrial dysfunction exists, which provides a challenge to separate cause and effects (1).

Vitamin A (VitA) metabolism is perturbed in obese and T2D patients and animal models (7–9). Dietary VitA attenuates oxidative stress and preserves mitochondrial function (10–12), and mitochondrial dysfunction is associated with the development of T2D (13). Our previous studies using the same experimental model identified a transcriptional program, by which VitA preserves cardiac energetic gene expression in DIO that might attenuate subsequent onset of mitochondrial dysfunction and diabetic cardiomyopathy (14). Liver, skeletal muscle, and kidney tissue each have a very high mitochondrial content (15) and are major contributors of T2D-associated complications, including adverse remodeling and mitochondrial dysfunction, which is similar to cardiac tissue (6).

However, the impact of VitA on mitochondrial energetics and the development of obesity- and T2D-associated organ damage in liver, skeletal muscle, and kidney is incompletely understood, which we aimed to investigate in the present study. To address this important question, we used our murine model of impaired liver retinoid levels and DIO, in which mice with germline deletion of *Lecithin retinol acyltransferase* (*Lrat* ^-/-^) (16) are subjected to VitA-deficient (VAD) normocaloric and VAD high fat diet (HFD) feeding (14). *Lrat*, a key enzyme of VitA metabolism, is responsible for the storage of VitA metabolites as retinyl esters. Global *Lrat* gene deletion decreases hepatic retinoid levels and *Lrat* ^-/-^ mice have impaired tissue retinoid levels in the absence of dietary VitA compared to WT controls (16–19). In the present study, the use of VAD diets minimizes VitA availability in *Lrat* ^-/-^ mice, both under normocaloric conditions and following HFD feeding (14). Using this model, we assessed the impact of VitA on mitochondrial energetics and organ damage in liver, skeletal muscle, and kidney that are critical for the development of T2D and that are major targets of T2D-associated complications.

## Materials and methods

### Animals

**Figure 1:**
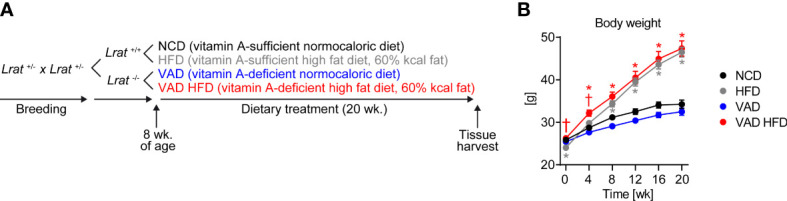
Experimental paradigm and increase in body weight independent of VitA following HFD feeding. **(A)** Mice with homozygous *Lrat* germline deletion (*Lrat* ^-/-^) and wildtype controls (*Lrat* ^+/+^) were obtained by breeding mice with heterozygous *Lrat* germline deletion (*Lrat* ^+/-^). Dietary treatment started at 8 weeks of age for a duration of 20 weeks total. **(B)** Body weight data are reported as mean values ± SE and represent a subgroup of a previously published animal cohort (14). Two-way ANOVA was performed to analyze differences by HFD feeding and VitA. Results of *post-hoc* analyses for each comparison are summarized by symbols as defined: # p<0.05 for HFD feeding, $ p<0.05 for VitA, and & p<0.05 for the interaction between HFD feeding and VitA. Body weight following 0 ($, &), 4 (#, &), 8 (#, &), 12 (#), 16 (#), and 20 (#) weeks of dietary feeding (n=17-18/group). *p < 0.05 vs. normocaloric diet same VitA availability, † p<0.05 vs. VitA sufficiency same caloric diet.

Previously, *Lrat* ^-/-^ mice (16) and wildtype littermate controls (*Lrat* ^+/+^) were examined for cardiac phenotypes and study (14). Specifically, mice were obtained by breeding mice with heterozygous *Lrat* germline deletion (*Lrat* ^+/-^) and were on a mixed C57BL/6J/129Sv genetic background. Dietary treatments started at 8 weeks of age for the duration of 20 weeks total (**Figure 1A**). Wildtype mice were fed with normal chow diet (13% kcal from fat; group indicated as “NCD”) or HFD (60% kcal from fat; group indicated as “HFD”). *Lrat* ^-/-^ mice were fed with normocaloric VAD diet (13% kcal from fat; group indicated as “VAD”) or VAD HFD (60% kcal from fat; group indicated as “VAD HFD”) (14). The composition of mouse diets is provided in **Supplemental Table 1**. All diets were purchased from Altromin (Lage, Germany). Animals were housed with free access to food and water and 12 h light/dark cycles. All studies were performed in male mice under random fed conditions. Experiments were performed in accordance with protocols approved by local state authorities (Niedersächsisches Landesamt für Verbraucherschutz und Lebensmittelsicherheit, protocol number: #17/2702). The current manuscript in contrast focused on non-cardiac organs, i.e., liver, kidney, and skeletal muscle, in these same animals to determine tissue-specific differences. HFD feeding increased body weight independent of VitA (**Figure 1B**). Body weight data presented in **Figure 1B** represent a subgroup of a previously published animal cohort that was used in our prior report (14).

### Measurement of mitochondrial oxygen consumption in isolated mitochondria

Immediately after harvest liver, quadriceps muscle, and kidneys were placed in ice-cold STE1 buffer (250 mmol/L sucrose, 5 mmol/L Tris/HCl, 2 mmol/L EGTA, pH 7.4). Tissues were minced and either incubated in 2.5 mL STE2 buffer (STE1 containing [wt/vol] 0.5% BSA, 5 mmol/L MgCl_2_, 1 mmol/L ATP, and 2.5 U/mL protease Subtilisin A) for 4 min (quadriceps muscle) or immediately proceeded to homogenization (liver and kidney tissue). All tissues were homogenized using a Teflon pistil in a Potter-Elvejhem homogenizer. Quadriceps muscle homogenates were further diluted with 2.5 mL STE1 buffer containing Complete Mini protease inhibitor cocktail (Roche, Mannheim, Germany), centrifuged at 8,000 g for 10 min, and the resulting pellet was resuspended in 4 mL STE1 buffer. Next, all tissues homogenates were centrifuged at 800 g for 10 min and supernatants were centrifuged at 8,000 g for 10 min. Pellets obtained from mitochondrial isolation were resuspended in 200 µL STE1 buffer each. State III oxygen consumption rates (V_ADP_) were determined in 300 µg of mitochondria by using a Clark-type oxygen electrode (Strathkelvin, North Lanarkshire, Scotland) with 20 µmol/L palmitoyl-carnitine (PC)/2 mmol/L malate or 10 mmol/L pyruvate/5 mmol/L malate as substrates as previously described (14).

### Hydroxyacyl-coenzyme A dehydrogenase (HADH) and citrate synthase (CS) enzyme activity assays

HADH and CS activity in liver, skeletal muscle, and kidney tissue were determined as previously described (20).

### Immunoblotting

Protein extraction and immunoblotting were performed as previously described (14). Proteins were resolved by SDS-PAGE, electrotransferred to PVDF membranes, and primary antibodies were incubated at 4°C overnight. Primary and secondary antibodies used are listed in **Supplemental Table 2**. Proteins were detected with horseradish peroxidase (HRP)-conjugated secondary antibodies (GE HealthCare, Chicago, IL, USA) and densitometric quantification was performed using the software Image J.

### Stereological and histopathological analysis of tissue sections

Tissues were fixed in 4% buffered formaldehyde, stored > 24 h prior to further processing, embedded in paraffin, and cut into 2 µm thick sections. Hematoxylin and eosin (H&E), Picrosirius red (PSR) and wheat germ agglutinin (WGA)/DAPI fluorescence staining were performed as previously described (14). Microscopy of H&E and PSR stains was performed using a BX43 light microscope (Olympus, Tokyo, Japan) and an Observer.Z1 fluorescence microscope (Zeiss, Wetzler, Germany) for WGA/DAPI stains. Liver fat content was assessed as area percentage of hepatocytes with macro- or microvesicular cytoplasmatic fat inclusions. Hepatic fibrosis was evaluated according to the ISHAK score (21), and liver sections were scored for steatosis, lobular inflammation, and hepatocyte ballooning according to the NAFLD activity score (NAS) (22). For immunohistological quantification of skeletal muscle fiber area, sections were deparaffinized, stained with WGA, and sections with WGA staining of the cellular membrane were selected for stereological quantification, which was performed using the AxioVision software (Zeiss, Wetzler, Germany). Kidney sections showing cortex and medulla were evaluated for histopathologic abnormalities with focus on the presence of tubular ischemia and necrosis, glomerular ischemia, microthrombi, arteriosclerosis and glomerulosclerosis. Sections were analyzed by an experienced pathologist blinded for the group of mice investigated.

### Quantitative RT-PCR analysis (qPCR)

RNA isolation from tissues, cDNA synthesis and quantitative real-time PCR were performed as previously described (14). Primers are listed in **Supplemental Table 3**.

### Statistical analysis

Data are presented as means ± standard error (SE). Data sets were analyzed by 2-way ANOVA for multi-group comparisons with Holm-Šídák’s *post hoc* analysis to determine significance levels by HFD feeding and VitA. Statistical analysis was performed using GraphPad Prism 8 software (GraphPad Software, Inc., La Jolla, CA). For all analyses, a p-value of <0.05 was considered significantly different.

## Results

### VitA regulates liver fibrotic gene expression and steatosis in DIO independent of altered mitochondrial metabolism

**Figure 2:**
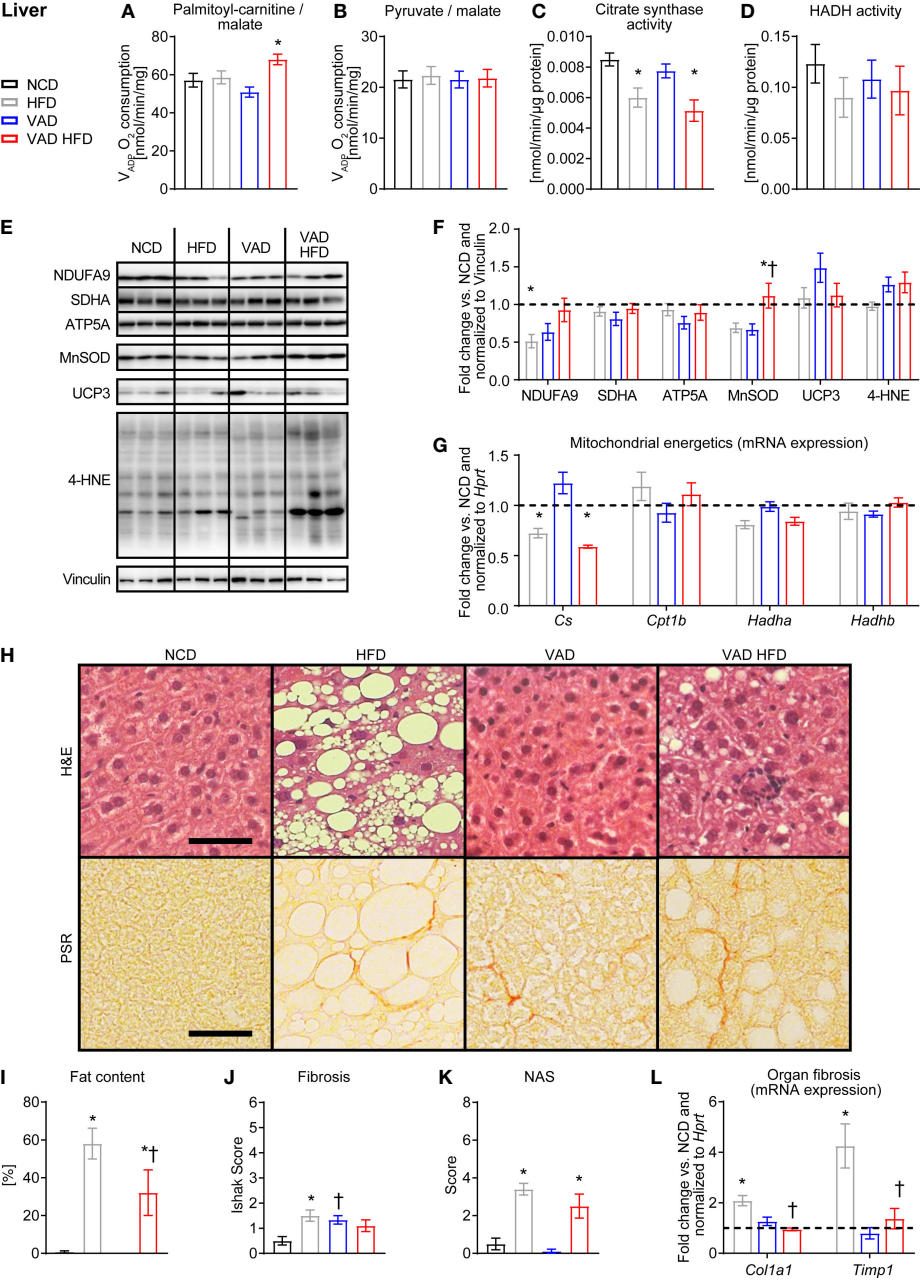
VitA modulates steatosis and fibrosis in liver tissue in DIO independent of mitochondrial function. Data are reported as mean values ± SE. Two-way ANOVA was performed to analyze differences by HFD feeding and VitA. Results of *post-hoc* analyses for each comparison are summarized by symbols as defined: # p<0.05 for HFD feeding, $ p<0.05 for VitA, and & p<0.05 for the interaction between HFD feeding and VitA. V_ADP_ of isolated liver mitochondria with **(A)** palmitoyl-carnitine (#, &) and **(B)** pyruvate each combined with malate as substrates (n=7-8). **(C)** Citrate synthase (#) and **(D)** HADH activity in liver tissue (n=6-8). **(E)** Representative immunoblots and **(F)** densitometric analysis of NDUFA9 (&), SDHA, ATP5A, MnSOD (&),UCP3, and 4-HNE ($) normalized to Vinculin. Data are presented as fold change relative to NCD (assigned as 1.0; dashed line), n=6. **(G)** mRNA expression of transcripts involved in mitochondrial energetics (*Cs:* #, &; *Hadha:* #). Data are presented as fold change relative to NCD and normalized to *Hprt* (assigned as 1.0; dashed line), n=7-8. **(H)** Representative H&E and PSR stains of liver sections (scale bars: 50 μm each). Quantification of **(I)** fat content (#), **(J)** fibrosis (&), and **(K)** NAFLD activity scores (NAS, #), n=9-10. **(L)** mRNA expression of transcripts involved in organ fibrosis (*Col1a1:* #, $, &; *Timp1:* #, $, &). Data are presented as fold change relative to NCD and normalized to *Hprt* (assigned as 1.0; dashed line), n=7-8. *p < 0.05 vs. normocaloric diet same VitA availability, † p<0.05 vs. VitA sufficiency same caloric diet.

PC/malate-supported V_ADP_ was not different between the HFD and VAD HFD groups and interestingly increased following HFD feeding under VAD conditions (**Figure 2A**). No difference in V_ADP_ with pyruvate/malate as substrates was detected between groups (**Figure 2B**). CS activity declined following HFD feeding independent of VitA (**Figure 2C**). Activity of HADH, a key enzyme of mitochondrial fatty acid oxidation (FAO), was not different between groups (**Figure 2D**). Protein abundance of selected respiratory chain subunits, MnSOD, UCP3, and 4 hydroxynonena (4-HNE) adducts was relatively unchanged between groups (**Figures 2E, F**). Consistent with the changes in activity, qPCR analysis showed decreased *Citrate synthase (Cs)* mRNA expression following HFD feeding independent of VitA status. Expression of selected FAO genes was not different between groups (**Figure 2G**). Histopathological analysis revealed reduced DIO-mediated hepatic steatosis in the VAD HFD group relative to the HFD group. Similarly, HFD feeding increased fibrosis relative to NCD, which was attenuated in the VAD HFD group (**Figures 2H–K** and **Supplemental Table 4**). HFD feeding increased mRNA expression of the pro-fibrotic transcripts *Tissue inhibitor of metalloproteinase 1 (Timp1)* and *Alpha-1 type I collagen (Col1a1)* (**Figure 2L**), which was attenuated in the VAD groups. Together, these data indicate that VitA mediates the pro-fibrotic transcriptional response, adverse remodeling, and steatosis in DIO independent of mitochondrial oxidative capacity.

### VitA status does not influence mitochondrial function and morphological structure following HFD feeding in skeletal muscle

**Figure 3:**
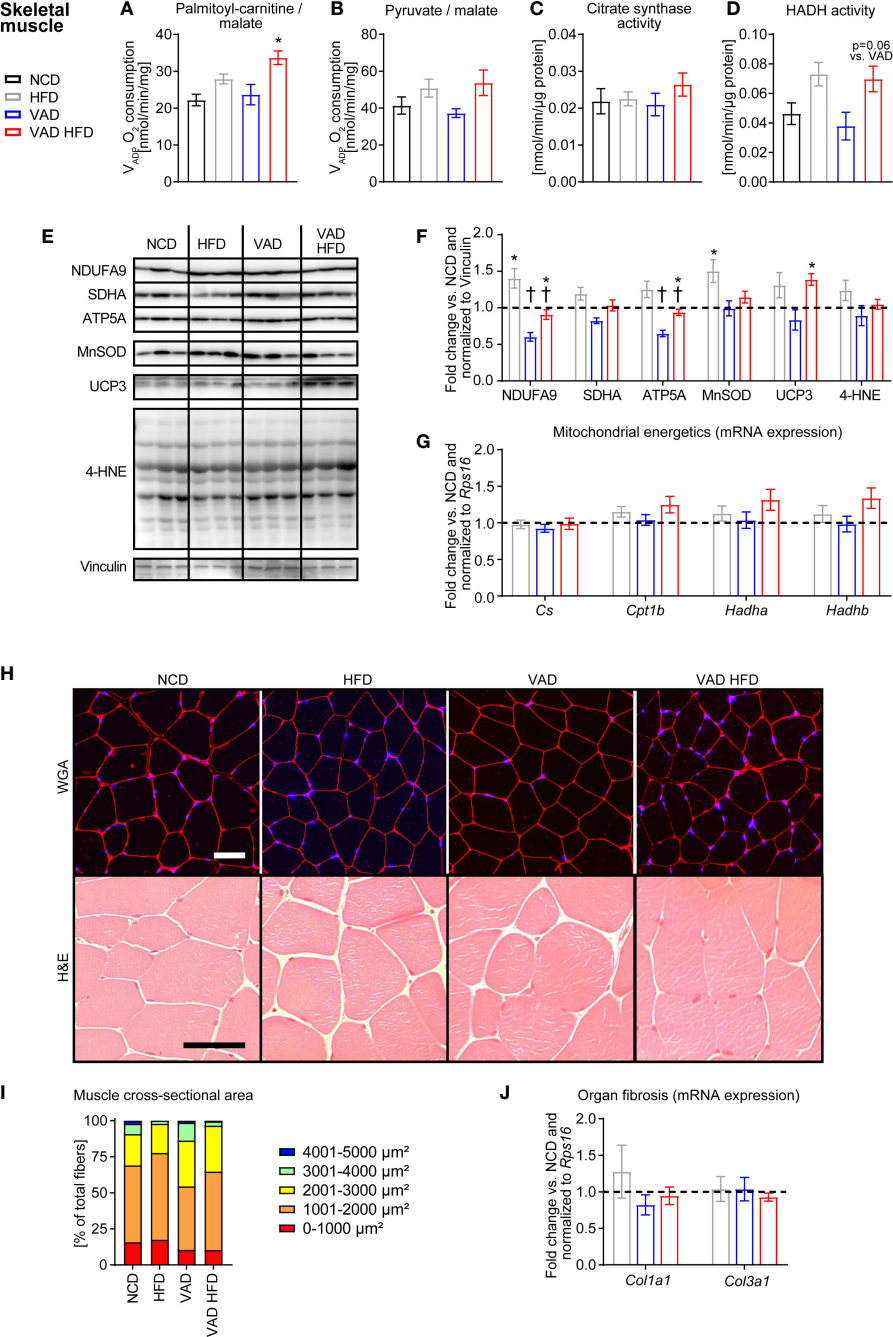
VitA does not impair skeletal muscle mitochondrial function and morphological structure in DIO. Data are reported as mean values ± SE. Two-way ANOVA was performed to analyze differences by HFD feeding and VitA. Results of *post-hoc* analyses for each comparison are summarized by symbols as defined: # p<0.05 for HFD feeding and $ p<0.05 for VitA. No significant effect for the interaction between HFD feeding and VitA was determined. V_ADP_ of isolated skeletal muscle mitochondria with **(A)** palmitoyl-carnitine (#) and **(B)** pyruvate (#) each combined with malate as substrates (n=6-8). **(C)** Citrate synthase and **(D)** HADH (#) activity in skeletal muscle (n=7-8). **(E)** Representative immunoblots and **(F)** densitometric analysis of NDUFA9 (#, $), SDHA (#, $), ATP5A (#, $), MnSOD (#),UCP3 (#), and 4-HNE normalized to Vinculin. Data are presented as fold change relative to NCD (assigned as 1.0; dashed line), n=6. **(G)** mRNA expression of transcripts involved in mitochondrial energetics presented as fold change relative to NCD and normalized to *Rps16* (assigned as 1.0; dashed line), n=8. **(H)** Representative WGA and H&E stains of skeletal muscle sections (scale bars: 50 μm each). **(I)** Mean cross-sectional area of skeletal muscle fibers presented as percentage of total fibers (# for 4001-5000 µm², n=5-6). **(J)** mRNA expression of transcripts involved in organ fibrosis presented as fold change relative to NCD and normalized to *Rps16* (assigned as 1.0; dashed line), n=8. *p < 0.05 vs. normocaloric diet same VitA availability, † p<0.05 vs. VitA sufficiency same caloric diet.

Like liver tissue, mitochondrial dysfunction in skeletal muscle alters whole body metabolism in T2D (23). V_ADP_ in skeletal muscle mitochondria was not different between the HFD and VAD HFD group with PC/malate as substrates but was increased in the VAD HFD relative to the VAD group (**Figure 3A**). Pyruvate/malate-supported V_ADP_ and CS activity were similar between groups (**Figures 3B, C**). HADH activity trended to increase following HFD feeding independent of VitA (HFD vs. NCD: + 58.0%, p=0.11; VAD HFD vs. VAD: + 84.8%, p=0.06; **Figure 3D**). Immunoblotting analysis revealed that VitA preserves protein abundance of NDUFA9 and ATP5A both under normocaloric and HFD feeding conditions (**Figures 3E, F**). Expression of selected transcripts involved in FAO and mitochondrial energetics was not changed between groups (**Figure 3G**). Histological and stereological analysis showed no gross morphological differences between groups. Mean cross-sectional area of skeletal muscle fibers presented as percentage of total fibers was evenly distributed between groups (**Figures 3H, I**). *Col1a1* and *Col3a1* mRNA expression was not different between groups (**Figure 3J**).

### VitA mediates the HFD-induced pro-fibrotic transcriptional response in kidney tissue independent of mitochondrial energetics

**Figure 4:**
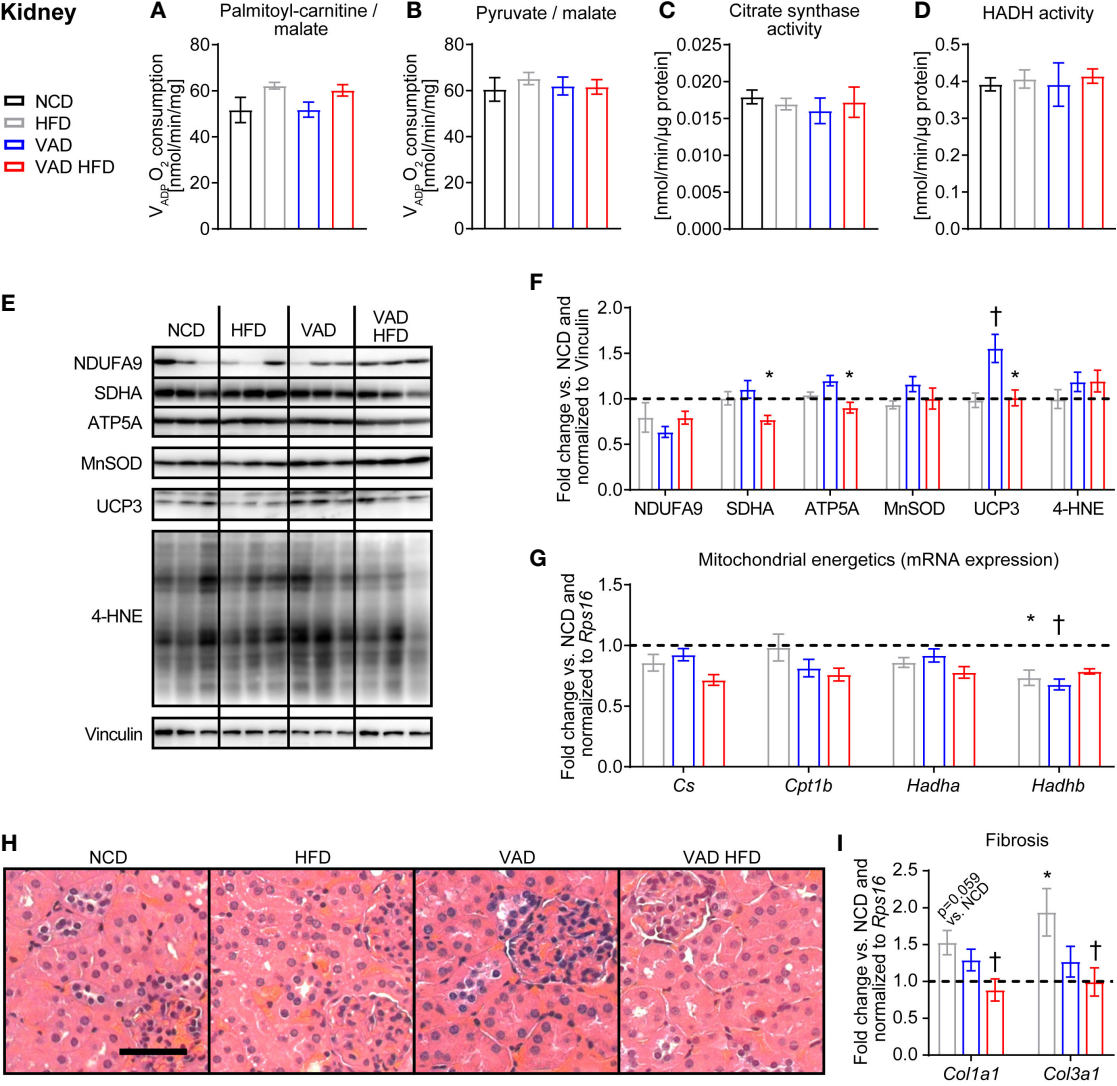
VitA mediates the pro-fibrotic transcriptional response in kidney tissue following HFD feeding independent of mitochondrial energetics. Data are reported as mean values ± SE. Two-way ANOVA was performed to analyze differences by HFD feeding and VitA. Results of *post-hoc* analyses for each comparison are summarized by symbols as defined: # p<0.05 for HFD feeding, $ p<0.05 for VitA, and & p<0.05 for the interaction between HFD feeding and VitA. V_ADP_ of isolated kidney mitochondria with **(A)** palmitoyl-carnitine (#) and **(B)** pyruvate each combined with malate as substrates (n=6-8). **(C)** Citrate synthase and **(D)** HADH activity in kidney tissue (n=7-8). **(E)** Representative immunoblots and **(F)** densitometric analysis of NDUFA, SDHA (&), ATP5A (&), MnSOD, UCP3 (#, $, &), and 4-HNE normalized to Vinculin. Data are presented as fold change relative to NCD (assigned as 1.0; dashed line), n=6. **(G)** mRNA expression of transcripts involved in mitochondrial energetics (*Cs:* #; *Cpt1b:* $; *Hadha:* #; *Hadhb:* $, &). Data are presented as fold change relative to NCD and normalized to *Rps16* (assigned as 1.0; dashed line), n=8. **(H)** Representative H&E stains of kidney sections (scale bar: 50 μm). **(I)** mRNA expression of transcripts involved in organ fibrosis (*Col1a1:* &; *Col3a1:* &). Data are presented as fold change relative to NCD and normalized to *Rps16* (assigned as 1.0; dashed line), n=8. *p < 0.05 vs. normocaloric diet same VitA availability, † p<0.05 vs. VitA sufficiency same caloric diet.

No difference in kidney mitochondria V_ADP_ with PC and pyruvate each combined with malate as substrates was detected between groups (**Figures 4A, B**). Similarly, HADH and CS enzymatic activity, protein abundance of selected respiratory chain subunits, UCP3, MnSOD, and 4-HNE adducts and mRNA expression of mitochondrial energetics genes were relatively unchanged between groups (**Figures 4C–G**). Histological analysis of kidney sections with focus on tubular ischemia and necrosis, glomerular ischemia, microthrombi, arteriosclerosis, and glomerulosclerosis showed a regular morphologic phenotype with no difference between groups (**Figure 4H**). Interestingly, the HFD-induced increase in the collagen subtype expression *Col1a1* and *Col3a1* was attenuated in the VAD HFD group (**Figure 4I**). These data indicate that VitA mediates the pro-fibrotic transcriptional response in DIO independent of mitochondrial capacity.

## Discussion

Mitochondrial dysfunction is associated with T2D (1, 2), and perturbations in VitA metabolism are observed in T2D patients and animal models (7–9, 24). The relationship between VitA metabolism and the development of mitochondrial dysfunction and end-organ damage in T2D is incompletely understood. The present study reveals that VitA regulates the pro-fibrotic transcriptional response and adverse organ remodeling in DIO in a tissue-specific manner that is independent of mitochondrial energetics and oxidative stress.

Our data show that pathological remodeling of liver tissue following HFD feeding is exacerbated by VitA signaling independent of mitochondrial capacity (**Figure 2**). Previous rodent studies investigated the impact of HFD feeding and T2D on liver mitochondrial energetics; however, the results have not been consistent. The parameters that contribute to the opposing results include the species, the duration of the dietary treatment, the genetic background, and the genetic modification of the models investigated. Hepatic retinol levels are inversely correlated with the severity of steatosis (8) and non-alcoholic fatty liver disease (NAFLD) in patients (25). One proposed mechanism for increased steatosis following excess caloric intake is hyperinsulinemia-mediated lipogenesis, which may contribute to hypertriglyceridemia (23, 26). Our previous report utilizing the same experimental protocol indicates impaired insulin sensitivity and glucose tolerance following HFD feeding as determined by insulin tolerance tests (ITT) and glucose tolerance tests (GTT), whereas VAD following normocaloric feeding had no effect (14). VAD attenuated HFD-induced development of T2D as indicated by attenuated glucose intolerance in GTT and a trend towards attenuated insulin resistance in ITT for the comparison VAD HFD relative to HFD (14). Importantly, basal serum insulin levels were not increased in the HFD group relative to the VAD HFD group (14). Together, these data indicate that VitA mediates HFD feeding-induced steatosis by mechanisms that are independent of circulating insulin levels, although effects on modulation of insulin action in hepatocytes remain to be explored.

Despite the well-characterized association between insulin resistance, T2D, and skeletal muscle mitochondrial dysfunction the cause and consequence remain a subject of discussion (27). Previous studies reported impaired skeletal muscle mitochondrial capacity in insulin resistant (3) and T2D patients (4), while others reported no impairment (27, 28). In the present study, HFD feeding did not affect skeletal muscle mitochondrial respiratory capacity under VitA-sufficient conditions (**Figures 3A, B**), which is like our results obtained from liver mitochondria (**Figures 2A, B**). Histological analysis and gene expression analysis for markers of organ fibrosis showed no difference between groups (**Figures 3H–J**), which reveals that VitA regulates tissue-specific organ remodeling and damage in DIO. Previous studies reported muscle atrophy following 38-weeks of HFD feeding in mice (29), which contrasts to the present study reporting no difference in mean cross-sectional area of skeletal muscle fibers between groups (**Figures 3H, I**). The different phenotypes reported following HFD feeding might emanate from the genetic background of the mice used and the duration of dietary treatment.

Mitochondrial dysfunction contributes to diabetic nephropathy (30). In the present study, no difference in mitochondrial capacity was detected between groups (**Figures 4A–D**). Mitochondria were isolated from total kidneys and activity of mitochondrial enzymes was determined in total kidney homogenates. Kidneys consist of different cell types and regions, i.e., medulla and cortex, that differentially adapt to the diabetic milieu (30). The protocol used for mitochondria isolation does not discriminate compartment and cell type-specific mitochondrial capacity, which might exist. Changes in mitochondrial bioenergetics precede histological changes in kidneys from type 1 diabetic rats (31). These data are in concert with the present study reporting preserved mitochondrial capacity following HFD and VAD in the absence of gross morphological changes (**Figure 4H**). The attenuated transcriptional response of collagen isoforms, i.e., *Col1a1* and *Col3a1*, in the VAD HFD group relative to the HFD group indicates that VitA signaling may contribute to a pro-fibrotic transcriptional program in DIO, which is like our observation in liver tissue (**Figures 2H–L**). This VitA-mediated transcriptional program may mediate subsequent kidney fibrosis and organ damage in DIO.

Numerous animal models investigated the relationship between dietary VitA intake and the pathophysiology and consequences of obesity and insulin resistance (32–36). VitA supplementation at a high dose of 129 mg/kg diet for the duration of two months, which corresponds to approximately 3 mg/kg body weight/day, attenuates body weight gain in obese WNIN/Ob rats relative to controls receiving diets containing 2.6 mg VitA/kg diet (32). Short-term retinoic acid supplementation decreases body weight and improves insulin sensitivity (34, 35), while feeding a VAD diet increases body weight and adiposity in mice (36). In the present study, the body weight increase following HFD feeding was not influenced by tissue levels of VitA (14). Moreover, mice subjected to VAD diets did not show characteristics of severe VAD, including alopecia, ataxia, or weight loss. All-*trans*-retinoic acid doses that are used in rodents studies to block or reverse accumulation of adipose tissue are typically very high and range up to 100 mg/kg body weight, which is equivalent to 6,000 mg/60 kg human being (37). All-*trans*-retinoic acid is used to treat patients with acute promyelocytic leukemia (APL) at a recommended dose of 45 mg/m^2^/d for adult patients. This is equivalent to approximately 80 mg/dose administered (38) and therefore much lower compared to doses that block or reverse adiposity in rodents. Importantly, about 1 in 4 patients with APL receiving all-*trans*-retinoic acid induction therapy develop “Differentiation Syndrome” (formerly known as “Retinoic Acid Syndrome”). Characteristics of this potential life-threatening side effect comprise unexplained fever, acute respiratory distress, capillary leak syndrome, and renal failure (39). Moreover, treatment of skin disease with 13-*cis* retinoic acid increases the risk of hyperlipidemia and metabolic syndrome (40). Patients with increased serum retinol levels exhibit an increased risk of hip fracture (41), which might be attributable to a crosstalk of VitA and vitamin D metabolism resulting in osteoporosis (42). Together, these serious effects defines the challenges inherent in using retinoic acid as a potential anti-obesity drug (37).

Previous studies investigated the impact of VitA on mitochondrial energetics and dynamics in different tissues. Dietary treatment of rats with VitA-deficient diets impairs function of mitochondria that were isolated from cardiac, but not from liver tissue (11, 12). Furthermore, the VitA metabolite all-trans retinoic acid (ATRA) increases dynamin-related protein (DRP1) levels and promotes mitochondrial fission in murine hearts (43). VitA mediates the expression of genes involved in oxidative phosphorylation as previously reported for the mitochondrial gene ATPase 6 in primary hepatocytes following stimulation with retinoic acid (44) and in cardiac tissue in DIO using the same experimental model used in the present study (14). Interestingly, mitochondrial gene expression was relatively preserved between groups in liver, skeletal muscle, and kidney in the present study. These data indicate that cardiac tissue is more susceptible to the loss of VitA on mitochondrial gene expression under HFD conditions. A potential mechanism for this observation is the mitochondrial content of cardiac tissue, which is the greatest across all tissues in mammals (15). Further studies are required to delineate the tissue-specific impact of VitA on mitochondrial energetics, both under basal conditions and in the context of superimposed stressors.

Limitations of our study include the use of adult mice at a relatively young age, which contrasts the onset of T2D-associated complications that are typically observed in older patients. Another limitation is that *Lrat* ^-/-^ mice were fed with VAD diets to study the relationship between VitA availability and manifestations of DIO (14). Therefore, we cannot dissociate the effect of the genetic modification of the mice investigated from the dietary treatment. It is important to note that hepatic all-*trans*-retinal and all-*trans*-retinol levels were nearly absent in *Lrat* ^-/-^ mice following feeding with VAD diets independent of dietary fat content (14). Hepatic retinoid levels represent whole body VitA status (45, 46). Even though not directly measured, these data indicate impaired tissue VitA availability in the VAD groups independent of dietary fat content, including skeletal muscle, and kidney. The profound impairment in VitA metabolite levels, which is accomplished by our combined transgenic and dietary approach, might only partially reflect the adaptations in patients following restricted dietary VitA intake and VitA levels. Mitochondrial capacity was determined by measuring V_ADP_, CS and HADH enzymatic activity. This experimental approach might not detect defects in mitochondrial energetics that might exist, and which might only be detected by very sophisticated measurements, including the measurement of mitochondrial membrane potential and ATP production that is required to directly assess coupling of mitochondrial ATP production to mitochondrial oxygen consumption.

In the present study, mitochondrial oxygen consumption was determined in isolated mitochondria. Previous studies used saponin-permeabilized tissue preparations for the measurement of mitochondrial oxygen consumption in skeletal muscle (47) and liver tissue (48, 49). A potential limitation of the isolated mitochondria technique is the disruption of the mitochondrial network and the potential enrichment of a mitochondrial population during the isolation process. Since acute changes in liver metabolism might not be detected in saponin-permeabilized liver tissue (49), we used isolated mitochondria for the measurement of oxygen consumption. It is of interest for future studies to determine the impact of HFD feeding and VitA on mitochondrial respiratory capacity in isolated mitochondria and in saponin-permeabilized tissue using a complementary experimental approach. Previous studies reported profound and tissue-specific differences in the mitochondrial proteome of type 1 diabetic mice (50) and VitA is a master regulator of transcriptional regulation (51, 52). Thus, another limitation is that we measured abundance of a limited number of transcripts and proteins involved in mitochondrial energetics in the present study. Our previous studies using the same experimental protocol identified that VitA preserves cardiac energetic gene expression in DIO; however, has no impact on cardiac remodeling, mitochondrial and contractile function following HFD feeding (14). This previous report supports the present study, which identifies a tissue-specific impact for VitA on adverse organ remodeling in DIO independent of mitochondrial function.

**Figure 5:**
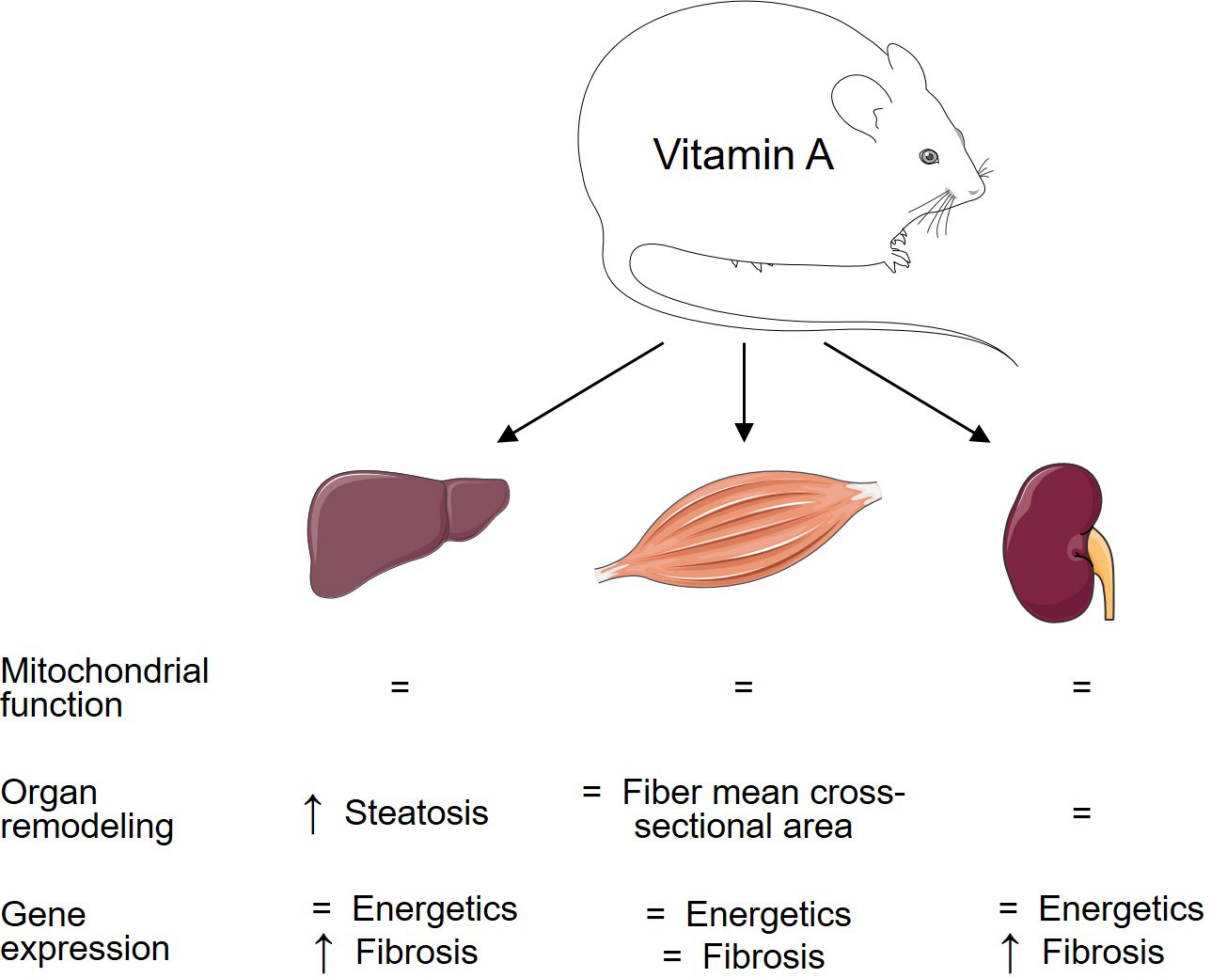
Tissue-specific impact of VitA on mitochondrial energetics and tissue remodeling in DIO. Changes in the HFD relative to VAD HFD group are summarized to visualize the relationship between VitA content and tissue responses to HFD feeding.

In summary, the present study identifies an unexpected role for VitA that regulates the pro-fibrotic transcriptional response and pathological remodeling in a tissue-specific manner in DIO that is independent of mitochondrial function and mitochondrial energetic gene expression (**Figure 5**). In contrast, our previous studies using the same experimental protocol identified a VitA-mediated transcriptional program that preserves cardiac energetic gene expression in DIO and that might attenuate subsequent mitochondrial dysfunction and diabetic cardiomyopathy (14). Thus, VitA mediates both, protective and adverse, effects on organ damage in DIO and T2D. The present study extends our knowledge on the complex and tissue-specific aspects of VitA metabolism in metabolic disease and highlights the importance of additional studies in this area of research prior to the use of VitA metabolites as potential anti-obesity drugs.

## Data availability statement

The original contributions presented in the study are included in the article/**Supplementary Material**. Further inquiries can be directed to the corresponding author.

## Ethics statement

The animal study was reviewed and approved by Niedersächsisches Landesamt für Verbraucherschutz und Lebensmittelsicherheit.

## Author contributions

CW, IS, LN, MS, and NF planned experiment, performed experiments and analyzed data. CR analyzed data. DJ, AW, EDA, JB, MK, and WB provided intellectual input for the project, interpreted data, and critically revised the manuscript. CR secured funding, conceived the study, prepared figures, and wrote the manuscript. All authors contributed to the article and approved the submitted version.
