# Supplementary material for: Vitamin A regulates tissue-specific organ remodeling in diet-induced obesity independent of mitochondrial function
Source: Front Endocrinol (Lausanne). 2023 Feb 20;14:1118751. doi: 10.3389/fendo.2023.1118751 (PMC9987331; doi:10.3389/fendo.2023.1118751)
Supplement: Supplementary file 1 [file DataSheet_1.pdf]

SUPPLEMENTARY MATERIAL

FOR

Vitamin A regulates tissue-specific organ remodeling in diet-induced obesity independent of mitochondrial function

Ivanna Shymotiuk[1, †], Natali Froese[1, †], Christopher Werlein[2], Lea Naasner[1], Malgorzata Szaroszyk[1], Mark P. Kühnel[2, 3], Danny D. Jonigk[2, 3], William S. Blaner[4], Adam R. Wende[5], E. Dale Abel[6], Johann Bauersachs[1], Christian Riehle[1, *]

[1] Department of Cardiology and Angiology, Hannover Medical School, Hannover, Germany
[2] Institute of Pathology, Hannover Medical School, Germany
[3] Biomedical Research in End-stage and Obstructive Lung Disease Hannover (BREATH), German Lung Research Centre (DZL), Hannover, Germany
[4] Department of Medicine, College of Physicians and Surgeons, Columbia University, New York, NY, USA
[5] Division of Molecular and Cellular Pathology, Department of Pathology, University of Alabama at Birmingham, Birmingham, AL, USA
[6] Department of Medicine, David Geffen School of Medicine and UCLA Health, Los Angeles, CA, USA

[†] These authors have contributed equally to this work and share first authorship.

Supplemental Table 1: *Composition of mouse diets*

Ingredient		Unit	NCD	HFD	VAD	VAD HFD
**Carbohydrate**		[% by calories]	**67.9**	**23.2**	**67.8**	**22.5**
**Protein**		[% by calories]	**20.1**	**16.5**	**20.1**	**16.6**
**Fat**		[% by calories]	**13.0**	**60.1**	**13.0**	**60.8**
**Amino acids**	Alanine	[g/kg]	2.53	11.68	2.53	11.76
	Arginine	[g/kg]	9.83	13.33	9.83	13.43
	Aspartic acid	[g/kg]	3.58	12.26	3.59	12.45
	Cystine	[g/kg]	3.20	1.41	3.20	1.43
	Glutamic acid	[g/kg]	23.67	29.68	23.69	30.18
	Glycine	[g/kg]	3.14	24.10	3.14	24.25
	Histidine	[g/kg]	5.28	3.42	5.28	3.48
	Isoleucine	[g/kg]	7.22	7.13	7.23	7.30
	Leucine	[g/kg]	14.76	7.48	14.77	7.52
	Lysine	[g/kg]	17.40	11.75	17.40	11.91
	Methionine	[g/kg]	7.22	4.32	10.69	4.41
	Phenylalanine	[g/kg]	7.17	7.04	7.18	7.16
	Proline	[g/kg]	12.76	20.46	12.77	20.67
	Serine	[g/kg]	5.27	8.12	5.27	8.25
	Threonine	[g/kg]	7.15	6.16	7.16	6.27
	Tryptophan	[g/kg]	1.98	1.25	1.98	1.29
	Tyrosine	[g/kg]	9.29	5.12	9.29	5.23
	Valine	[g/kg]	3.30	7.49	3.30	7.64
**Fatty acids**	C4, Butyric	[g/kg]		4.19		4.36
	C7, Caproic	[g/kg]		3.68		3.83
	C8, Caprylic	[g/kg]		1.27		1.32
	C10, Capric	[g/kg]		2.62		2.72
	C12, Lauric	[g/kg]	0.05	3.13	0.05	3.25
	C14, Myristic	[g/kg]	0.10	13.90	0.10	14.34
	C16, Palmitic	[g/kg]	2.50	56.56	2.50	56.67
	C17, Margaric	[g/kg]	0.10	0.43	0.10	0.43
	C18, Stearic	[g/kg]	1.35	37.17	1.35	37.49
	C20, Arachidic	[g/kg]	0.05	3.79	0.05	3.79
	C22, Behenic	[g/kg]	0.15		0.15	
	C24, Lignoceric	[g/kg]	0.25		0.25	
	C16:1, Palmitoleic	[g/kg]	0.15	8.90	0.15	8.99
	C18:1, Oleic	[g/kg]	13.50	111.56	13.50	112.45
	C-20:1, Eicosaenoic	[g/kg]	0.15	1.71	0.15	1.71
	C22:1, Erucic	[g/kg]	0.05	1.75	0.05	1.82
	C18:2, Linoleic	[g/kg]	28.50	22.12	28.50	22.17
	C18:3, Linolenic	[g/kg]	0.15	3.13	0.15	3.16

Ingredient		Unit	NCD	HFD	VAD	VAD HFD
**Vitamins**	Biotin	[mg/kg]	0.20	0.20	0.20	0.20
	Choline chloride	[g/kg]	1.01	1.00	1.01	1.00
	Folic acid	[mg/kg]	10.00	10.02	10.01	10.02
	Nicotinic acid	[mg/kg]	50.17	50.03	50.17	50.03
	Pantothenic acid	[mg/kg]	50.11	50.02	50.11	50.02
	Vitamin A	[I.E./kg]	15,000	15,000		
	Vitamin B1	[mg/kg]	20.04	20.01	20.04	20.01
	Vitamin B2	[mg/kg]	20.32	20.06	20.32	20.06
	Vitamin B6	[mg/kg]	15.23	15.21	15.03	15.01
	Vitamin B12	[mg/kg]	0.04	0.03	0.04	0.03
	Vitamin C	[mg/kg]	20.00	20.00	21.00	21.00
	Vitamin D3	[I.E./kg]	500.00	500.00	500.00	500.00
	Vitamin E	[mg/kg]	180.40	150.07	180.40	150.07
	Vitamin K3	[mg/kg]	10.20	10.00	10.20	10.00
**Nutrients**	Aluminum	[mg/kg]	3.71	2.37	3.74	2.45
	Calcium	[g/kg]	9.31	7.21	9.54	7.28
	Chlorine	[g/kg]	3.63	3.93	3.63	4.00
	Cobalt	[mg/kg]	0.15	0.09	0.15	0.09
	Copper	[mg/kg]	5.75	3.32	5.65	3.32
	Fluorine	[mg/kg]	4.17	3.03	4.17	3.03
	Iodine	[mg/kg]	0.51	0.27	0.45	0.27
	Iron	[mg/kg]	178.58	104.43	178.61	104.42
	Magnesium	[g/kg]	0.68	0.60	0.67	0.60
	Manganese	[mg/kg]	100.89	58.94	100.89	58.95
	Molybdenum	[mg/kg]	0.20	0.18	0.20	0.18
	Phosphorus (digested)	[g/kg]	7.20	4.45	7.21	4.49
	Phosphorus (total)	[g/kg]	7.52	5.35	7.53	5.41
	Potassium	[g/kg]	7.09	6.58	7.17	6.67
	Selenium	[mg/kg]	0.33	0.17	0.33	0.17
	Sodium	[g/kg]	2.49	1.67	2.50	1.67
	Sulfur	[g/kg]	2.79	1.42	2.79	0.68
	Zinc	[mg/kg]	29.30	15.24	29.31	15.24
**Supplements**	Cholesterol	[mg/kg]		211.85		211.85
	Chondroitin	[g/kg]		31.20		31.20
	Glucosamine	[g/kg]		66.00		66.00
	Inositol	[mg/kg]	111.00	101.93	111.00	101.93

NCD, normal chow diet; HFD, high fat diet; VAD, vitamin A-deficient diet; VAD HFD, vitamin A-deficient high fat diet.

Supplemental Table 2: *Antibodies used for immunoblotting*

**Antigen**	**Company**	**Catalog number**
4-HNE	Abcam, Cambridge, MA, USA	46545
ATP5A	Abcam, Cambridge, MA, USA	14748
MnSOD	BD Biosciences, San Jose CA, USA	611580
NDUFA9	Abcam, Cambridge, MA, USA	14713
SDHA	Abcam, Cambridge, MA, USA	14715
UCP3	Abcam, Cambridge, MA, USA	180643
Vinculin	Santa Cruz Biotechnology, Santa Cruz, CA	7649
Anti-goat	Jackson ImmunoResearch, West Grove, PA, USA	305-035-045
Anti-mouse	GE HealthCare, Chicago, IL, USA	NA934V
Anti-rabbit	GE HealthCare, Chicago, IL, USA	NXA931V

Supplemental Table 3: *Primers used for quantitative real-time PCR analysis*

**Gene Name**<br>**Gene Sequence of forward and**<br>**reverse primers (5'→ 3')**<br>**GenBank Accession Number**
Carnitine palmitoyltransferase 1b (Cpt1b)<br>TGCCTTTACATCGTCTCCAA<br>AGACCCCGTAGCCATCATC<br>NM_009948.2
Citrate synthase (Cs)<br>CTCACAGTGGGGTGCTGCT<br>CCCAGTCTCCCATTTTACCC<br>NM_026444.4
Collagen, type I, alpha 1 (Col1a1)<br>TGGTGAACGTGGTGTACAAGGT<br>CAGTATCACCCTTGGCACCAT<br>NM_007742.4
Collagen, type III, alpha 1 (Col3a1)<br>AAGGCTGAAGGAAACAGCAA<br>TGGGGTTTCAGAGAGTTTGG<br>NM_009930.2
Hydroxyacyl-CoA dehydrogenase trifunctional multienzyme complex subunit alpha (Hadha)<br>TCAGGAGGGCTCAAAGAATAA<br>GAAAGCCAAGCCCAAAGAC<br>NM_178878.3
Hydroxyacyl-CoA dehydrogenase trifunctional multienzyme complex subunit beta (Hadhb)<br>GCCAACAGACTGAGGAAGGA<br>ACACTGGCAAGGCTGGATT<br>NM_001289798.1
Hypoxanthine guanine phosphoribosyl transferase (Hprt)<br>GGACCTCTCGAAGTGTTGGATAC<br>GCTCATCTTAGGCTTTGTATTTGGCT<br>NM_013556.2
Ribosomal protein S16 (Rps16)<br>TGCTGGTGTGGATATTCGGG<br>CCTTGAGATGGGCTTATCGG<br>NM_013647.2
Tissue inhibitor of metalloproteinase 1 (Timp1)<br>TCTGAGCCCTGCTCAGCAA<br>AACAGGGAAACACTGTGCACAC<br>NM_001044384.1

Supplemental Table 4: *Analysis of liver sections according to NAFLD Activity Score (NAS)*

Endpoint	NCD	HFD	VAD	VAD HFD
Steatosis [Score] #, $	0.20 ± 0.13	2.20 ± 0.25 *	0.00 ± 0.00	1.20 ± 0.39 *†
Lobular inflammation [Score] #	0.00 ± 0.00	0.50 ± 0.17	0.00 ± 0.00	0.50 ± 0.22
Hepatocyte ballooning [Score] #	0.30 ± 0.21	0.70 ± 0.21	0.11 ± 0.11	0.80 ± 0.13
NAS #	0.50 ± 0.31	3.40 ± 0.31 *	0.11 ± 0.11	2.50 ± 0.64 *

Data are reported as mean values ± SE, n=9-10. Two-way ANOVA was performed to analyze differences by HFD feeding and VitA. Results of post-hoc analyses for each comparison are summarized by symbols as defined: # p<0.05 for HFD feeding and $ p<0.05 for VitA. No significant effect for the interaction between HFD feeding and VitA was determined. * p<0.05 vs. normocaloric diet same VitA availability, † p<0.05 vs. VitA sufficiency same caloric diet.
